# Differential Role of CD318 in Tumor Immunity Affecting Prognosis in Colorectal Cancer Compared to Other Adenocarcinomas

**DOI:** 10.3390/jcm14145139

**Published:** 2025-07-19

**Authors:** Bhaumik Patel, Marina Curcic, Mohamed Ashraf Eltokhy, Sahdeo Prasad

**Affiliations:** 1 Department of Immunotherapeutic and Biotechnology, Texas Tech University Health Science Center, Abilene, TX 79601, USA; mcurcic@ttuhsc.edu (M.C.); mohamed.eltokhy@ttuhsc.edu (M.A.E.); 2 R&D Life Sciences LLC., 13707 66th Street N, Largo, FL 33773, USA; spbiotech@gmail.com

**Keywords:** CDCP1/CD318, SRC, PKCδ, TGFβ1, T and NK cells, overall survival

## Abstract

**Background/Objectives**: CD318 (also known as CDCP1) is a transmembrane protein that is overexpressed in many cancers and contributes to tumor progression, invasion, and metastasis by activating SRC family kinases through phosphorylation. Emerging evidence also suggests that CD318 plays a role in modulating the tumor immune microenvironment, although its precise mechanism in tumor progression is still not well understood. **Methods**: To investigate this, we analyzed the expression and immune-related functions of CD318 using the publicly available data from The Cancer Genome Atlas (TCGA) across colorectal adenocarcinoma (COAD), cervical squamous cell carcinoma (CESC), lung adenocarcinoma (LUAD), and pancreatic adenocarcinoma (PAAD). **Results**: All four cancers exhibited a high level of CD318 expression. Notably, in CESC, LUAD, and PAAD, plasmin-mediated cleavage of CD318 leads to phosphorylation of SRC and protein kinase C delta (PKCδ), which activates HIF1α and/or p38 MAPK. These downstream effectors translocate to the nucleus and promote the transcriptional upregulation of TGFβ1, fostering an immunosuppressive tumor microenvironment through Treg cell recruitment. In contrast, this signaling cascade appears to be absent in COAD. Instead, our analysis indicate that intact CD318 in COAD interacts with the surface receptors CD96 and CD160, which are found on CD8^+^ T cells and NK cells. **Conclusions**: This interaction enhances cytotoxic immune responses in COAD by promoting CD8^+^ T cell and NK cell activity, offering a possible explanation for the favorable prognosis associated with high CD318 expression in COAD, compared to the poorer outcomes observed in CESC, LUAD, and PAAD.

## 1. Introduction

CD318 (CUB Domain-Containing Protein 1), a 140 kD cell surface transmembrane glycoprotein, has been implicated in various cellular processes, including tumor progression, metastasis, and interaction within the tumor immune microenvironment. CD318 contains three CUB (complement protein subcomponents C1/r, urchin embryonic growth factor, and bone morphogenic protein 1) domains within the extracellular region and a hexalysine stretch within the cytoplasmic region [1]. The intracellular hexalysine stretch region of CD318 has been known to interact with SRC kinase, leading to increased metastasis properties in melanoma cancers [2]. Moreover, cleaved intracellular CD318 is phosphorylated and activated by SRC kinase, enabling it to form a complex with activated integrin β1. This interaction subsequently triggers the FAK/PI3K/Akt motility signaling pathway, promoting early tumor dissemination [3]. On the contrary, the CUB domain of the extracellular region has been explored less, and its interaction with CD6 on immune cells is scarcely described.

Normally, CD318 is present on many epithelial cells [4], some hematopoietic cells [5], and mesenchymal stem cells [6] in multiple organs. Previous studies have shown that CD318 is widely expressed across various cancers, with its expression levels correlating with increased tumor aggressiveness and metastatic potential [2,7,8,9]. Recent developments identified CD318 as a ligand for CD6, suggesting its involvement in autoimmune diseases that affect the central nervous system and the synovial lining of joints [10]. In addition, soluble CD318 is chemo-attractive to T cells and levels of soluble CD318 are selectively and significantly elevated in the synovial fluid from patients with rheumatoid arthritis and juvenile inflammatory arthritis [10]. Moreover, CD318 expression in myeloid dendritic cells suppresses the proliferation of autoreactive T cells, a well-known targeted self-antigen in Type 1 Diabetes, demonstrating the involvement of the CD318/CD6 axis in the immunopathogenesis of inflammation [11].

The interaction of CD318 and CD6 has been involved in the tumor microenvironment, mainly in suppressing the cytotoxic effect of T and NK cells. Ruth et al. demonstrated that blocking this interaction using UMCD6 enhances the killing of breast cancer cells through distinct effects on CD8+ T and NK cells [9]. Similarly, the blockade of the CD6-CD318 interaction by itolizumab increases the cytotoxicity of CD8+ T and NK cells over CD318+ tumor lines, reverses the NKG2A/NKG2D ratio, and increases granzyme B and IFNγ production. In addition, it regulates immune responses by upregulating well-known immune checkpoint proteins PD-1 and CTLA-4 on lymphocytes, enhancing CD318+ tumor cell cytotoxicity [12]. However, the immune participation of CD318 is still emerging as a new avenue to study in multiple cancer models, and its impact on prognosis and targeted therapy remains underexplored. Although elevated expression of CD318 in various malignancies is known, systematic comparisons of its immune-regulatory effects and prognostic significance across distinct adenocarcinomas have not been conducted. Therefore, in this article, we have investigated the expression of CD318, its association with immune cells in the tumor microenvironment, and prognostic significance from The Cancer Genome Atlas (TCGA) and various publicly available databases. Using various analytical methods, we identified the genomic and functional networks and pathways associated with the expression of CD318 and its involvement in the immune microenvironment in various cancers such as colorectal adenocarcinoma (COAD), cervical squamous cell carcinoma (CESC), lung adenocarcinoma (LUAD), and pancreatic adenocarcinoma (PAAD). Thus, our findings reveal new insights into CD318 and its immune interactions, which can be utilized to propose further biological confirmation for human cancer diagnosis, prognosis, and treatment.

## 2. Materials and Methods

### 2.1. STRING

STRING database provides known and predicted protein–protein interactions, which include direct and indirect associations from computational predictions and are aggregated from other databases. Functional enrichments include Biological process, Molecular function, Cellular component, and KEGG pathways. STRING analysis for CD318 was conducted using this database.

### 2.2. GEPIA2

GEPIA2 is an advanced web server for extensive expression profiling and interactive analysis from the TCGA data portal and the GTEx database, respectively. The single gene module of GEPIA2 was used to study the mRNA expression levels of CD318 in cancer tissues and normal tissues. Kaplan–Meier curves were used to study the survival outcomes in which TCGA patient data were separated into high and low expression of CD318 groups.

### 2.3. Human Protein Atlas (HPA)

The human protein atlas is a program that has mapped all the human proteins across all major tissues and organs in the human body, as well as the expression of proteins in cancer with their impact on survival. Protein expression of CD318 in normal and multiple cancers was checked and relative protein expression values were obtained from the Human Protein Atlas. Relative protein expression provides a quantitative measure of protein abundance across various cancers and normal tissue from the patients’ proteomics database of the Cancer Genome Atlas [13].

### 2.4. TIMER 2.0

The correlation between CD318 expression and tumor-infiltrating immune cells was systematically assessed across multiple cancer types using Tumor Immune Estimation Resource 2.0 (TIMER 2.0), a comprehensive web-based resource for immune infiltration analysis. Specifically, the association of CD318 with infiltrating CD8+ T cells and natural killer (NK) cells was evaluated. Spearman correlation was used for analysis, with a *p*-value < 0.05 considered significant.

### 2.5. TISIDB

The Tumor–Immune System Interactions and Drug Bank database, an integrated web-based resource for analyzing tumor–immune system interactions, was utilized to investigate the correlation between CD318 and various immune-related components, including lymphocytes, immunomodulators, and chemokines. Particularly, the correlation of CD318 with TGFβ1, CD96, CD160 and abundance of Treg cells, CD8+ T cells, and NK cells with CD318 expression.

### 2.6. ClusPro

Protein–protein docking analyses were performed using the ClusPro 2.0 web server to predict the potential binding interfaces between CD318 and immune cell receptors CD96 and CD160. ClusPro is widely recognized for its performance in CAPRI (Critical Assessment of Predicted Interactions) benchmarking and physically grounded scoring functions. The 3D structures of CD318, CD96, and CD160 were retrieved from the AlphaFold as it provides full-length, high-confidence structural predictions for human proteins. In ClusPro docking, CD318 was used as a receptor and CD96 and CD160 were used as ligands for inputs. Docking simulations were conducted using four distinct scoring paradigms available in ClusPro: (1) Balanced: equal contributions from electrostatics, hydrophobicity, and van der Waals forces. (2) Electrostatic-favored: prioritizing electrostatic potential at the interface. (3) Hydrophobic-favored: emphasizing hydrophobic contacts and interface burial. (4) Van der Waals + electrostatics (VdW + Elec): a hybrid model combining dispersion and polar interactions. Upon generation of thousands of potential docking confirmations, which are automatically clustered by structural similarity, top clusters were ranked based on cluster size (indicating structural convergence and stability) and weighted energy score (the more negative values indicate more favorable predicted binding). For each receptor–ligand pair, the center and lowest energy structures from top five clusters were analyzed and mapped using 5 Å cutoff to identify probable interactions. Cross-validation was conducted by comparing predicted interfaces across all four scoring models. Consistent identification of core interacting residues across models was used to establish confidence in predicted binding interfaces.

### 2.7. Data Availability

The datasets utilized in this study are publicly accessible and were obtained from the following sources: The Cancer Genome Atlas (TCGA), the Genotype-Tissue Expression (GTEx) project funded by the Common Fund, and the Search Tool for the Retrieval of Interacting Genes/Proteins (STRING) database.

### 2.8. Statistical Significance

Data obtained from various analytical tools utilize multiple statistical methods. GEPIA2 web server and TISIDB database utilize log-rank test (Mantel-Cox test) for survival analysis and one-way ANOVA (F-test) for expression analysis. The gene set variation analysis (GSVA) was used to infer the relative abundance of immune cells, while TIMER 2.0 data is computed by the Wilcoxon test and annotated by *p* values. Relative protein expression plots are generated using GraphPad Prism, version 10.2.3, Statistical significance has been obtained using an unpaired *t*-test and *p*-values < 0.05 were considered statistically significant.

## 3. Results

### 3.1. Involvement of CD318 in Biological Pathways

**Figure 1:**
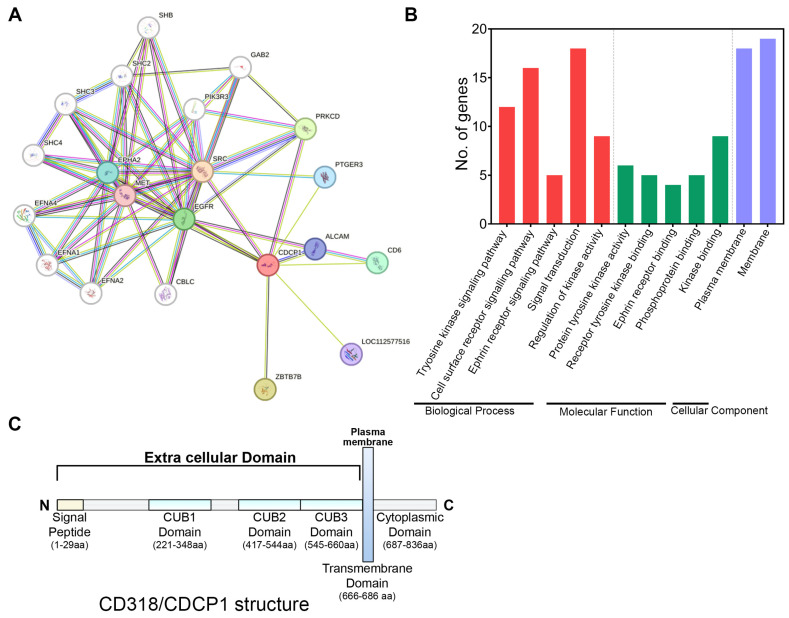
Gene ontology, pathway enrichment, and structure of CD318. (**A**) String analysis of CD318 showing direct and indirect interactions of proteins with CD318. (**B**) Gene Ontology (GO) enrichment analysis of CD318 involves three aspects: Biological process, Molecular function, and Cellular compartment. (**C**) Structure of CD318 showing signal peptide, extracellular CUB domains, and intracellular cytoplasmic domain.

CD318 is known to be involved in the SRC activation pathway, thereby modulating cell growth, survival, and metastasis in cancer. To explore its broader functional network, we performed a STRING analysis, which identifies predicted and known interactions between proteins. This analysis revealed that CD318 is associated with several key signaling and immune-related molecules, including SRC, EGFR, PRKCD, PTGER3, ALCAM, and CD6 (Figure 1A). These associations support the involvement of CD318 in both oncogenic signaling and immune-related pathways. The interaction of CD318 with SRC has been well studied, and has shown that the CD318/SRC axis contributes to the development of multiple cancers, including breast [14], melanoma [2], and lung [15]. Given that the STRING analysis revealed predicted interactions between tumor-associated CD318 and immune-related proteins such as CD6 and PRKCD (Figure 1A), we further investigated the functional context of these associations using a Gene Ontology (GO) analysis. The GO enrichment results highlighted three major categories: (i) Biological processes, including tyrosine kinase signaling (e.g., via SRC) and cell surface receptor signaling pathways, suggesting potential interactions between the extracellular domain of CD318 and immune receptors such as CD6; (ii) Molecular functions, indicating involvement in kinase activity and phosphoprotein binding; and (iii) Cellular components, showing the predominant localization of CD318 at both the extracellular and intracellular regions of the plasma membrane (Figure 1B, Table 1). The structure of CD318 consists of a signal peptide and three CUB domains, which can interact with immune cells for immune activation/suppression or can be utilized for plasmin cleavage to activate the intracellular kinase (Figure 1C). Overall, these findings suggest that CD318’s structure and biological function include involvement in tyrosine kinase signaling pathways such as the SRC pathway, epidermal growth factor receptor (EGFR) pathway, and cell surface receptor signaling pathways including the T Cell receptor (TCR) signaling pathway.

### 3.2. High Expression of CD318 in Multiple Cancers

**Figure 2:**
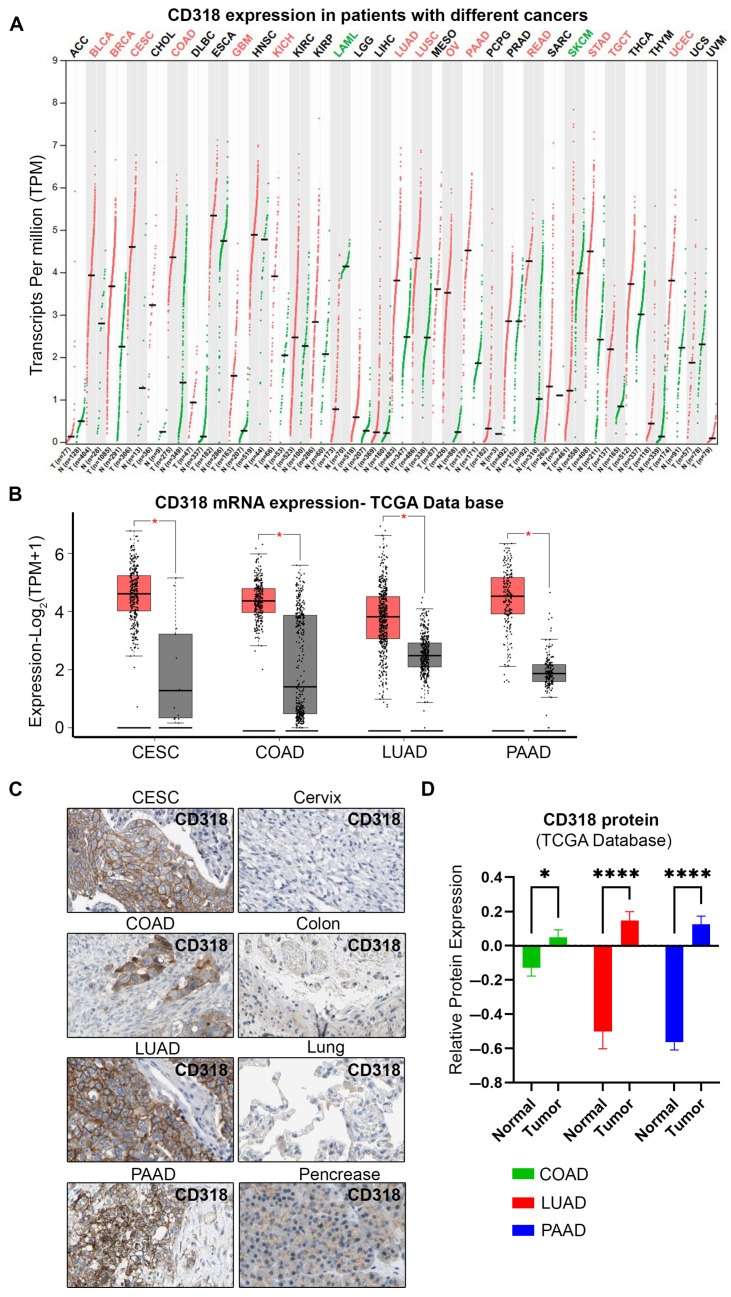
mRNA and protein expression of CD318 in multiple cancers. (**A**) Analysis of CD318 mRNA expression in various cancers compared to their corresponding normal tissues using TCGA database and GEPIA2 tool. (**B**) Significantly elevated mRNA expression of CD318 in CESC, COAD, LUAD, and PAAD cancer patients compared to normal tissue. (**C**) Immunohistochemistry images of CD318 protein in CESC, COAD, LUAD, and PAAD and their normal tissue from the human protein atlas. (**D**) Quantification of relative protein expression of CD318 protein in COAD, LUAD, and PAAD vs. normal tissue. Each dot represents an expression of the sample, where * indicates *p* ≤ 0.05, and **** indicates *p* ≤ 0.0001, via *t*-test. T indicates tumor and N indicates normal tissue.

To explore the transcriptional levels of CD318 expression in multiple cancers, we used the TCGA cancer database and GEPIA (Gene Expression Profiling Interactive Analysis) web server database. Our search revealed that various cancers such as bladder (BLCA), breast (BRCA), cervical (CESC), colorectal (COAD), kidney (KICH), lung (LUAD, LUSC), ovarian (OV), pancreatic (PAAD), and stomach cancers (STAD) have significantly increased CD318 expression. In contrast, Acute Myeloid Leukemia (LAML) and skin cutaneous melanoma (SKCM) cancers express significantly lower transcripts of CD318 compared to normal tissue (Figure 2A). Further quantification of CD318 expression showed that cervical cancer (CESC), colorectal cancer (COAD), lung adenocarcinoma (LUAD), and pancreatic cancer (PAAD) express significantly higher transcripts per million (TPM) compared to normal tissue, with 24.23, 19.72, 12.31, and 21.32 TPM median expression values, respectively, and significant *p* values (Figure 2B, Table 2). We further analyzed CD318 expression at the protein level using immunohistochemistry (IHC) data from the Human Protein Atlas (HPA) database. The HPA IHC analysis supported our findings from TCGA and GEPIA, revealing strong CD318 expression in tumor tissues of CESC, COAD, LUAD, and PAAD, with markedly lower expression in corresponding normal tissues (Figure 2C). The qualitative IHC data were confirmed by a relative protein expression analysis, showing significantly elevated levels in COAD, LUAD, and PAAD (Figure 2D). Thus, the high levels of CD318 expression in CESC, COAD, LUAD, and PAAD led us to further investigate its role in tumor progression and metastasis as well as in the immune microenvironment.

### 3.3. CD318 Is Associated with SRC and FAK Proteins in CESC, LUAD, and PAAD but Not in COAD

**Figure 3:**
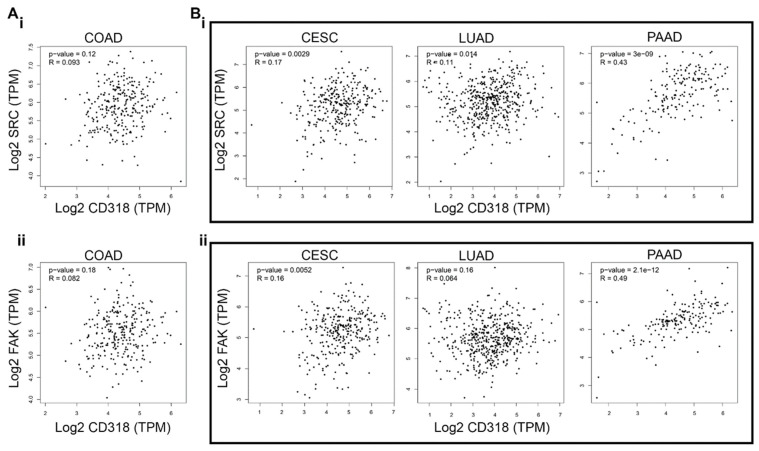
Correlation between CD318 and SRC/FAK in COAD, CESC, LUAD, and PAAD. (**A**) Non-significant mRNA correlation of SRC (**i**) and FAK (**ii**) with CD318 in COAD. (**B**) Significant mRNA correlation of SRC (**i**) and FAK (**ii**) with CD318 in CESC, LUAD, and PAAD.

**Figure 4:**
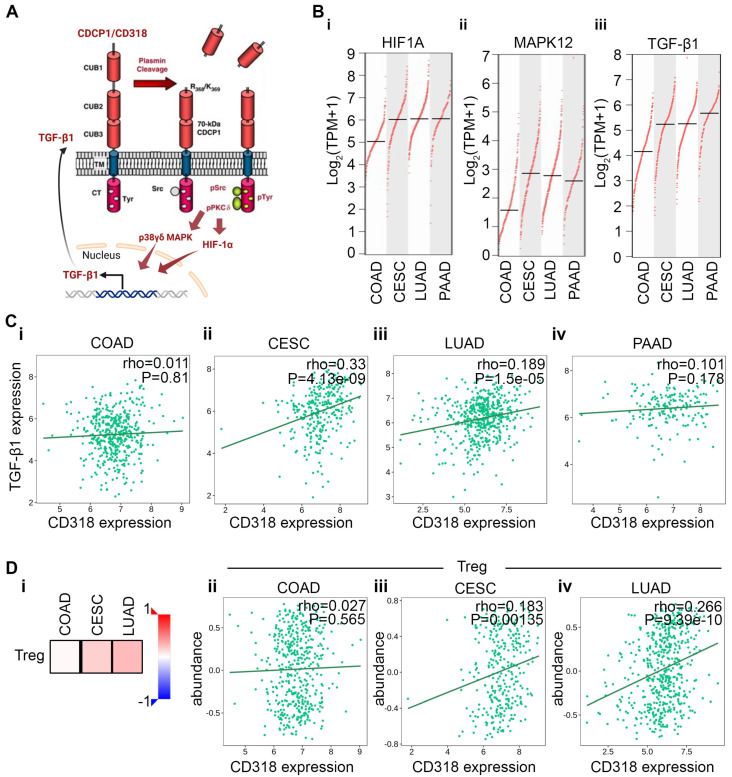
Plasmin cleavage of CD318 phosphorylates PKCδ, increasing TGFβ1-mediated immune suppression in CESC and LUAD but not in COAD. (**A**) Diagram showing that the plasmin cleavage of CD318 phosphorylates PKCδ, activates HIF1α, and phosphorylates p38 MAPK, leading to increased transcription of TGFβ1. (**B**) mRNA expression of HIF1α (**i**), MAPK12 (**ii**), and TGFβ1 (**iii**). (**C**) Correlation between CD318 and TGFβ1 in COAD (**i**), CESC (**ii**), LUAD (**iii**), and PAAD (**iv**) using TISIDB. (**D**) (**i**) Heatmap of correlation between CD318 and Treg cells, and correlation of abundance of Treg with CD318 expression in COAD (**ii**), CESC (**iii**), and LUAD (**iv**) using TISIDB.

Research has shown that CD318’s intracellular domain interacts with SRC [2] (Figure 4A), and SRC as well as FAK have been associated with solid tumor metastasis due to their ability to promote the epithelial–mesenchymal transition. Therefore, a strong correlation between increased FAK/SRC expression/phosphorylation and the invasive phenotype in human tumors has been established [16]. In addition, the interaction of CD318 and SRC/FAK may promote anchorage-independent cell growth and metastasis in cancers [17], particularly in pancreatic cancer [18,19] and lung cancer [20,21]. To validate this connection, we ran a correlation analysis of SRC and FAK with CD318 in four highly CD318-expressing cancers, such as COAD, CESC, LUAD, and PAAD. We found a significant positive correlation of CD318 with SRC and FAK in CESC, LUAD, and PAAD (Figure 3B(i,ii)), but no correlation in COAD (Figure 3A(i,ii)). These strong correlations in CESC, LUAD, and PAAD indicate the involvement of SRC/FAK-mediated tumorigenesis through CD318, however, there might be other pathways involved in COAD.

### 3.4. Differential Immune Suppression Through PKCδ/HIF-1α/TGFβ1 Axis in CESC, LUAD, and PAAD Compared to COAD

Murakami et al. and others have shown that plasmin cleavage of CD318 leads to the phosphorylation of Src and PKCδ [15,22]. Phosphorylated PKCδ subsequently increases the expression and transcriptional activity of HIF-1α [23]. In addition, pPKCδ phosphorylates MAPK kinase, promoting the nuclear translocation of both proteins and enhancing the transcription of TGFβ1 [24,25,26] (Figure 4A). We examined the expression levels of HIF1α, MAPK12, and TGFβ1 and found that their transcription varies across cancer types, with COAD showing the lowest expression and CESC, LUAD, and PAAD exhibiting significantly higher mRNA levels (Figure 4B(i–iii)). These data suggest higher plasmin cleavage of CD318, and subsequent activation and phosphorylation of HIF1α and MAPK12 and increased transcription of TGFβ1 in CESC, LUAD, and PAAD. In contrast, the expression of these proteins in COAD is not elevated when compared to normal tissue (Figure S1). We decided to explore this further using a TISIDB analysis. Our analysis revealed a non-significant correlation between the expression of TGFβ1 and CD318 in COAD, while CESC and LUAD showed a significant positive correlation (Figure 4C(i–iv)). Activation of the TGF-β signaling pathway can elicit either tumor-suppressing or tumor-promoting effects in a cell–cell context-dependent manner [27]. TGFβ1 plays an immune suppressive role across various cancers [28,29]. Moreover, TGFβ1 promotes CD4+ T cell differentiation into Treg cells through the maintenance of FOXP3 expression [29,30]. Therefore, we investigated the correlation between CD318 and Treg cells. The Spearman correlation analysis using the TISIDB database revealed a significant positive correlation between CD318 and the abundance of Treg cells in the tumor microenvironment of LUAD and CESC (*p* = 9.39 × 10^−10^ and *p* = 0.00135, respectively) vs. a non-significant correlation in COAD, as expected (Figure 4D(i–iv)). Although PAAD showed a negative correlation between CD318 and the abundance of Treg cells, typically, PAAD is considered a cold tumor with low immunogenicity, resulting in a limited number of tumor-specific antigens that the immune system can recognize. These results suggest the presence of PKCδ/HIF-1α/TGFβ1-mediated immune suppression in CESC, LUAD, and PAAD, while COAD cancers do not result in this immune suppression. Moreover, COAD might have an inhibition in the plasmin cleavage of CD318.

### 3.5. T and NK Cell-Mediated Cytotoxicity Through CD160 and CD96 Association and Interaction of CD318 in COAD Cancer

**Figure 5:**
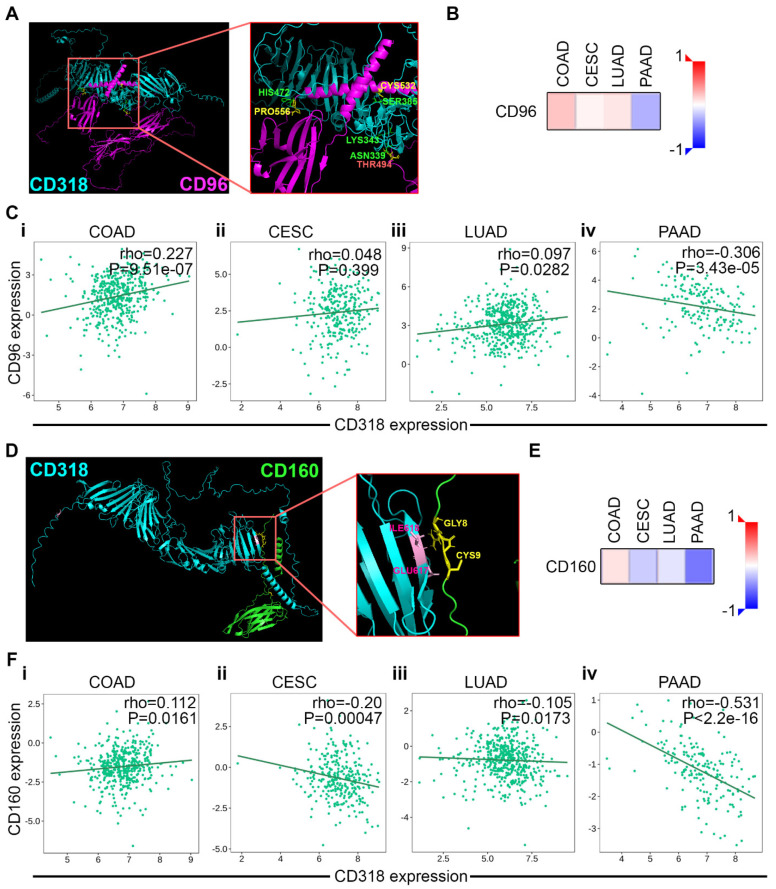
Prediction of interaction and association of CD96 and CD160 with CD318 in COAD. Docking site identification between CD318 and CD96 (**A**) and CD318 and CD160 (**D**) using ClusPro. Heatmap of correlation between CD318 and CD96 (**B**) and CD160 (**E**). Spearman correlations of CD318 expression with CD96 expression (**C**) and CD160 expression (**F**) in COAD (**i**), CESC (**ii**), LUAD (**iii**), and PAAD (**iv**) using TISIDB.

CD96, also known as Tactile, is primarily expressed on T cells and NK cells and enhances CD8+ T cell activation [31]. Additionally, CD96 is structurally similar to CD6, and works as a cell adhesion molecule like CD6. Recent evidence suggests that CD96 plays an important role in immune responses and positively collaborates with other checkpoint members [32]. Additionally, elevated CD96 expression correlated with CD8 expression and infiltration of NK cells [33]. With the expectation of an interaction between CD96 and CD318, we explored the crystal structures of CD318 and CD96 and analyzed their potential interactions using a docking server. Protein–protein docking between CD318 (receptor) and CD96 (ligand) was performed using the ClusPro web server under four scoring models: balanced, electrostatic-favored, hydrophobic-favored, and Van der Waals + electrostatic. Our results indicate that the balanced docking model generated strong clustering, with highly favorable energy scores: −1425.1 for the center model and −1503.6 for the lowest-energy model. Four key interactions were identified: (i) CD318 (ASN339) at CUB1 with CD96 (THR494, THR495), (ii) CD318 (SER385) with CD96 (CYS532), (iii) CD318 (HIS472) at CUB2 with CD96 (PRO556), and (iv) CD318 (LYS343) at CUB1 with CD96 (PRO555). Notably, these possible interactions are located on the extracellular domain at the CUB1 and/or CUB2 regions of CD318, suggesting a strong possible interaction of CD96 with CD318, similar to CD6 (Figure 5A). Our in-depth analysis using the TISIDB database revealed a significant positive correlation between CD96 and CD318 expressions in COAD (Figure 5C(i)). In contrast, CESC and LUAD have not shown a significant association, while PAAD had a significant negative association (Figure 5C(ii–iv)). These findings are further supported by a heatmap of Spearman correlations between CD318 and CD96 across COAD, CESC, LUAD, and PAAD (Figure 5B).

CD160 is a co-stimulatory receptor that enhances the activation and proliferation of NK cells and CD8+ T cells, particularly during chronic viral infections, rather than being solely responsible for T cell activation [34]. Sun et al. observed reduced expression of CD160 on intra-tumoral NK cells, and patients with lower CD160+ cell densities within tumors exhibited worse disease and a higher recurrence rate [35]. Moreover, CD160+ NK cells exhibited functional activation, high IFNγ production, and higher NK-mediated immunity [35]. This led us to look for possible interactions and associations of CD160 with CD318. The ClusPro docking results of these two proteins revealed strong and consistent binding predictions in balanced and hydrophobic-favored models. The balanced model produced a top cluster with 30 members and a weighted energy score of −1374.4 and the hydrophobic-favored model yielded the most stable configuration, with 50 members and a score of −1957.5, indicating a highly favorable interaction interface. The key residues at the predicted interface included GLU617, ILE618, and GLN22 on CD318 interacting with GLY6, ARG7, GLY8, CYS9, and PRO24 on CD160. These residues are localized to the extracellular CUB2 and signal peptide domains of CD318 and the N-terminal Ig-like domain of CD160 (Figure 5D). Similar to CD96, we observed a significant association of CD318 with CD160 in COAD (Figure 5F(i)) while CESC, LUAD, and PAAD expressed a negative association (Figure 5F(ii–iv)), which was supported by a heatmap of Spearman correlations (Figure 5E). Together, these docking and expression data support a model in which CD318 forms stable extracellular interactions with both CD96 and CD160 specifically in COAD.

**Figure 6:**
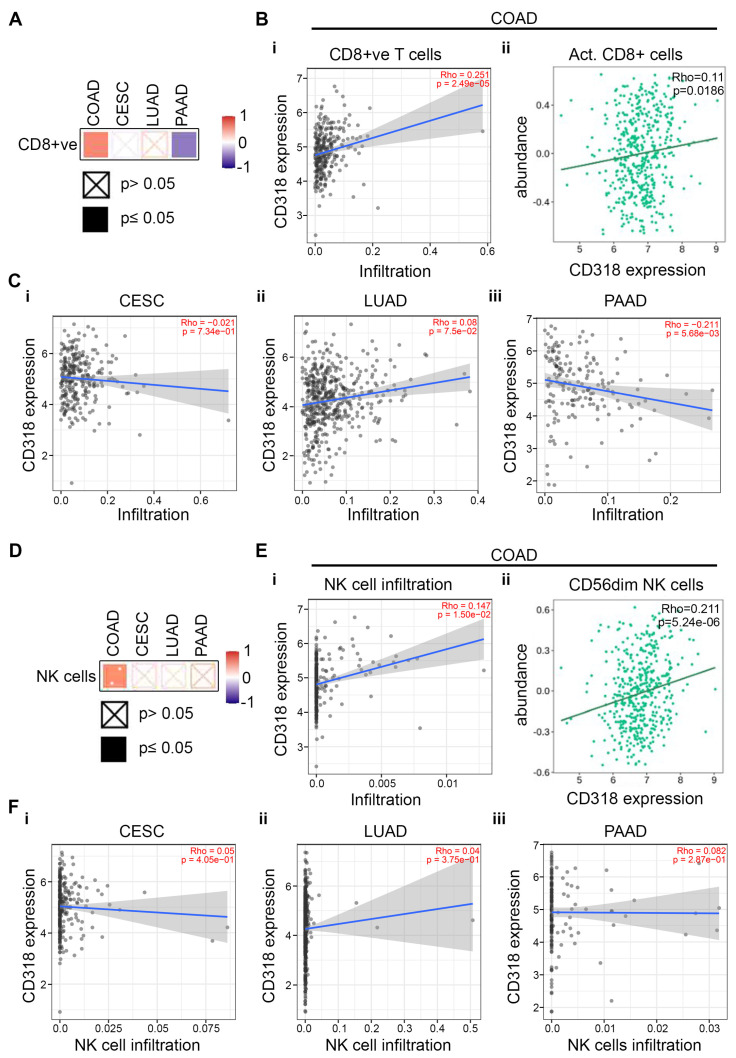
Correlation between CD318 and infiltrating CD8+ T cells and NK cells. The heatmap of the correlation of CD318 with CD8+ T cells (**A**) and NK cells (**D**) in COAD, CESC, LUAD, and PAAD. (**B**) (**i**) Scatter plot of relationship between infiltrating CD8+ T cells and CD318 expression (**ii**) Abundance of activated CD8+ T cells with CD318 expression in COAD. (**C**) Relationship between infiltrating CD8+ T cells and CD318 expression in CESC (**i**), LUAD (**ii**), and PAAD (**iii**). (**E**) (**i**) Analysis of infiltrating NK cells with CD318 expression and (**ii**) abundance of CD56^dim^ NK cells with CD318 expression in COAD. (**F**) Analysis of infiltrating NK cells with CD318 expression in CESC (**i**), LUAD (**ii**), and PAAD (**iii**).

Research indicates that co-expression and association of CD96 and CD160 with CD318 influence the regulation and activation of CD8+ T cells and NK cells, thereby affecting cytotoxic responses and anti-tumor immunity [31,35]. To explore this hypothesis further, we look for infiltration of CD8+ T cells and NK cells in COAD, CESC, LUAD, and PAAD. Using Timer 2.0 Web data server from the TCGA database, we identified that COAD cancer shows significantly higher levels of CD8+ T cell infiltration (*p* = 1.74 × 10^−6^) (Figure 6B(i)) compared to CESC, LUAD, and PAAD (Figure 6C(i–iii)). The heatmap of Spearman’s correlations showed similar results (Figure 6A). The abundance of CD8+ T cells also correlated with the expression of CD318 in COAD (Figure 6B(ii)), supporting the claim of higher CD8+ T cells in COAD cancer. Similar to CD8+ T cells, we observed significantly higher NK cell infiltration with respect to CD318 expression in COAD (Figure 6E(i)), but not in CESC, LUAD, and PAAD (Figure 6F(i–iii)). This result was supported by the heatmap expression of NK cells (Figure 6D). The cytotoxic activity of NK cells can be evaluated by expression of CD56, further subtyped as the CD56^dim^ NK subset and CD56^bright^ NK subset. CD56^dim^ NK subset cells are mature and considered as highly cytotoxic as they express higher levels of granzyme B and perforin [36,37]. The increased abundance of highly cytotoxic CD56^dim^ NK cells with CD318 expression in COAD confirms the presence of a higher cytotoxic response and anti-tumor immunity in COAD (Figure 6E(ii)).

### 3.6. Difference in Immune Response Affects the Prognostic Significance of CD318 in COAD Compared to CESC, LUAD, and PAAD

**Figure 7:**
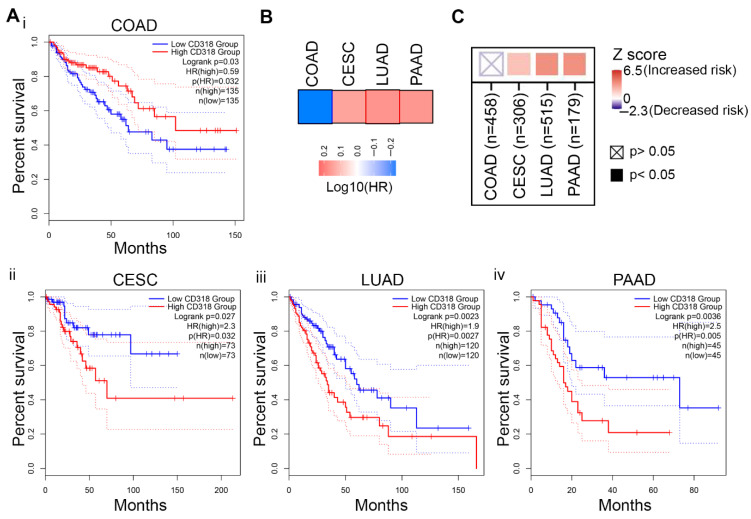
Prognostic analysis of CD318 in COAD, CESC, LUAD, and PAAD. (**A**) Analysis of overall survival (OS) with regards to CD318 expression using Kaplan–Meier in COAD (**i**), CESC (**ii**), LUAD (**iii**) and PAAD (**iv**) using the GEPIA 2 dataset. (**B**) Survival contribution of CD318 gene in COAD, CESC, LUAD, and PAAD cancers, estimated using Mantel–Cox test. (**C**) Analysis of clinical relevance of CD318 expression across four cancer types by Z score using Timer 2.0 analytical tool.

Elevated mRNA and protein expression of CD318 and its influence on the immune microenvironment may significantly affect survival outcomes. Thus, we performed a TISCH 2.0 analysis to examine the correlation between CD318 expression and patient survival. The results indicate that CD318 expression significantly impacts survival outcomes, as reflected by the calculated hazard ratios. PAAD and LUAD have an increased risk (*p* < 0.05), while COAD indicates a decreased risk (*p* ≥ 0.05) (Figure S2). Using the GEPIA web server tool, we performed an overall survival (OS) analysis in CD318 highly expressed COAD, CESC, LUAD, and PAAD cancers. Kaplan–Meier curve indicated a link between the high expression of CD318 and poor outcomes in CESC, LUAD, and PAAD, with significant *p* values of 0.027, 0.0023, 0.0036, respectively (Figure 7A(ii–iv)). In contrast, COAD had favorable outcomes though it had high CD318 expression (*p* = 0.03) (Figure 7A(i), Table 2), which suggests the influence of the cytotoxic immune microenvironment. Moreover, this finding was supported by the Mantel–Cox test, which evaluates the survival impact of gene expression and revealed poorer outcomes in CESC, LUAD, and PAAD, while indicating improved outcomes in COAD (Figure 7B). To further check the clinical relevance of CD318 across these cancers, we performed the Timer 2.0 gene analysis. The outcome module indicates an increased risk associated with higher expression of CD318 in CESC, LUAD, and PAAD but not in COAD, as represented by a Z score (Figure 7C). Thus, differences in the outcome of overall survival in COAD with elevated levels of CD318 strongly suggest the involvement of the immune interactions of CD318 and their influence on prognosis in COAD.

## 4. Discussion

CD318 has been implicated in tumor invasion and metastasis. A knockdown of CD318 or inhibition of its phosphorylation through Src-targeted therapy effectively abrogated anoikis resistance, migration, and invasion induced by activated Ras [38]. Moreover, activation of MMP2 and secretion of MMP9, in a model of Ras-induced invasion, was found to be regulated through the induction of phosphorylated CD318 [38]. CD318 facilitates the disruption of β-catenin and E-cadherin interactions, promoting the translocation of these proteins to the nucleus, resulting in tumor growth and metastases in preclinical models [39]. Thus, elevated levels of CD318 are associated with an increased occurrence of resistance and metastasis involving multiple mechanisms in various cancer models. Our results support this claim by demonstrating the high expression of CD318 and increased association with SRC/FAK, which is a known oncoprotein involved in tumor invasion and metastasis. While most cancers are aligned with this mechanism, COAD has also shown additional immune-related interactions and associations of CD318.

CD318 is phosphorylated at tyrosine residues in the intracellular domain by SRC family kinases and recruits PKCδ to the plasma membrane through tyrosine phosphorylation-dependent association with the C2 domain of PKCδ. This, in turn, induces a survival signal in an anchorage-independent condition [22]. Phosphorylation of PKCδ activates several survival pathways such as ERK1/2, NF-κB, STAT1, B-cell homeostasis, T cell activation and proliferation, and maintenance of Treg cells through TGFβ1 production via MAPK and HIP1α activation [40]. Moreover, in advanced cancer, TGFβ1 promotes tumor progression through EMT, immune evasion, angiogenesis, ECM remodeling, and T cell differentiation [41]. Our data indicate that in COAD, CD318 does not follow canonical plasmin cleavage and subsequent activation of the SRK/PKCδ/HIF-1α/TGFβ1 axis as observed in CESC, LUAD, and PAAD. In contrast, it leads to decreased TGFβ1 expression and inhibits the recruitment of Treg and CD4+ T cell differentiation [29,42].

Given its interaction with immune cells, CD318 has emerged as a potential target for stimulating the immune system to enhance anti-tumor immunity. Particularly, the CD6–CD318 axis, where CD6, which is expressed on T and NK cells, interacts with CD318 expressed on tumor cells. Disruption of the CD6–CD318 interaction with UMCD6, an anti-CD6 monoclonal antibody, augments lymphocyte cytotoxicity and prolongs survival [43]. Huang et al. have studied the association of CD318 with the cervical cancer immune microenvironment and stated that CD318 modulates the immune microenvironment of cervical cancer through the inhibition of the JAK-STAT pathway in T cells by binding to CD6 [44]. They also demonstrated that phytoestrogen 8-prenylnaringenin (8PN) suppressed cervical cancer effectively through the inhibition of CD318 [44]. Additionally, inhibition of CD318 by 8PN was utilized in lung cancer cells. Specifically, 8PN therapy was used to overcome CD318-mediated resistance to epidermal growth factor receptor (EGFR) tyrosine kinase inhibitors (TKIs). Treatment with 8PN reduced CD318 protein levels and malignant behavior by increasing IL-6 and IL-8 expression, which in turn promoted neutrophil infiltration and enhanced cytotoxicity against lung cancer cells [45].

**Figure 8:**
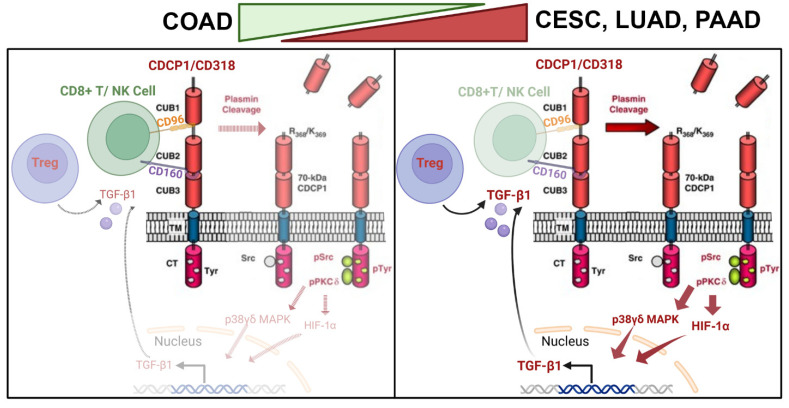
Diagram of mechanism of CD318 involving immune cells in COAD vs. CESC, LUAD, and PAAD. COAD has CD8+ T/NK cell-mediated anti-tumor immune response, whereas CESE, LUAD, and PAAD have TGFβ1/Treg cell-mediated immune suppression.

Itolizumab, a humanized anti-CD6 antibody, enhanced the cytotoxic activity of CD8^+^ T cells and NK cells against CD318-expressing tumor cell lines. It also reversed the NK2A/NK2D ratio and promoted an increased release of granzyme B and IFN-γ [12]. Furthermore, Fukuchi et al. have demonstrated potential therapeutic interventions resulting from targeting CD318. In their study, they produced function-blocking human anti-CD318 antibodies using human scFv phage display libraries, which effectively prevented the metastasis of human cancer cells in both mouse and chick embryos [46]. Ji et al. found that elevated expression of BRD4 and CBP/p300 is associated with increased CD318 expression. They demonstrated that NEO2734, a dual inhibitor targeting both BRD4 and p300, suppresses CD318 transcription and its downstream signaling pathways, thereby inhibiting proliferation and metastasis in castration-resistant prostate cancer cells [47]. The presence of CD318 on immune cells has also been explored. Do J et al. reported CD318 expression in a subpopulation of CD318+ myeloid dendritic cells (mDCs), whereas the other peripheral blood populations were negative for CD318. These CD318+ DCs suppressed the proliferation of autoreactive T cells specific for GAD65, a well-known targeted self-antigen in Type 1 Diabetes (T1D) [11]. Upon exploration of CD318 interactions with immune proteins, we found that CD96 and CD160 might be interacting with CD318 in COAD cancer, leading to the accumulation of CD8+ T Cells and CD56^dim^ highly cytotoxic NK cells in the tumor microenvironment. This proposed interaction led us to draw a model that differentiates COAD cancer from the other three, CESC, LUAD, and PAAD, cancers (Figure 8). This proposed novel mechanism might be the reason CD318 predicts a better outcome for COAD cancers in contrast to CESC, LUAD, and PAAD cancers.

## 5. Conclusions

Overall, we have evaluated the expression and association of CD318 with the SRC family and immune cells in various cancers. Through a comprehensive analysis of a publicly available database, we revealed that CD318 has divergent immunological consequences, correlating with a favorable prognosis in colorectal cancer (COAD) but unfavorable outcomes in cervical, lung, and pancreatic adenocarcinomas. These findings underscore a critical concept in precision oncology: what is beneficial in one cancer context may be detrimental in another. Thus, CD318 exemplifies how a uniform biomarker or therapeutic target can yield contrasting outcomes depending on tumor type and immune contexture, reminding us that in cancer immunotherapy, one size does not fit all.

### Future Directions and Limitations

These observational data were obtained from the patient database of The Cancer Genome Atlas (TCGA). Despite being one of the largest databases, the TCGA database exhibits demographic limitations as it under-represents certain ethical and racial groups compared to the US population. With incomplete or missing information about the treatment history, stage of disease, or follow-up details, the TCGA database also has clinical limitations. Moreover, the high prevalence of pre-existing and comorbid conditions among cancer patients may influence clinical outcomes. Therefore, experimental validation using in vitro cell culture models, as well as additional in vivo studies, is necessary to confirm these findings and to further elucidate the role of CD318 and its interactions with immune cells in the context of cancer.

## Figures and Tables

**Table 1:** Biological pathways and matching proteins involved in CD318 networks based on KEGG pathway.

Pathway	Description	Matching Proteins
hsa01521	EGFR tyrosine kinase inhibitor resistance	EGFR, MET, SRC
hsa04015	Rap1 signaling pathway	EGFR, MET, EPHA2, SRC
hsa04520	Adherens junction	EGFR, MET, SRC
hsa05120	Epithelial cell signaling in Helicobacter pylori infection	EGFR, MET, SRC
hsa04915	Estrogen signaling pathway	EGFR, PRKCD, SRC
hsa04360	Axon guidance	MET, EPHA2, SRC
hsa04510	Focal adhesion	EGFR, MET, SRC
hsa05205	Proteoglycans in cancer	EGFR, MET, SRC
hsa04014	Ras signaling pathway	EGFR, MET, EPHA2

**Table 2:** Statistical analysis of the expression of CD318 in multiple cancers along with % of five-year survival data and significance.

Cancer Type	TPM Median Expression	*p* Value	% of Five-Year Survival with High Expression	% of Five-Year Survival with Low Expression	Protein Expression	Significance	Condition
Breast cancer	11.75	0.049	81	93	Low/medium	Not prognostic	
**Cervical cancer**	**24.23**	**0.011**	**52**	**75**	**Medium**	**Potential prognostic**	**Unfavorable**
**Colorectal cancer**	**19.72**	**0.0052**	**79**	**52**	**High/medium**	**Potential prognostic**	**Favorable**
Endometrial cancer	13.47	0.47	68	56	Low/medium	Not prognostic	
Glioma	2.02	0.98	0	13	Low	Not prognostic	
Head and neck cancer	27.76	0.008	42	52	Low/medium	Not prognostic	
Liver cancer	0.23	0.014	44	63	Low	Not prognostic	
**Lung adenocarcinoma**	**12.31**	**9.2 × 10^−6^**	**27**	**46**	**Medium**	**Validated prognostic**	**Unfavorable**
Lung squamous cell carcinoma	18.51	0.027	39	52	Medium	Not prognostic	
Melanoma cancer	1.75	0.058	29(3 yr)	57(3 yr)	Low	Not prognostic	
Ovarian cancer	9.87	0.005	24	37	Low/medium	Not prognostic	
**Pancreatic cancer**	**21.32**	**0.00028**	**16**	**53**	**Medium**	**Potential prognostic**	**Unfavorable**
Prostate cancer	6.38	0.069	100	97	Low/medium	Not prognostic	
Renal cancer	4.59	0.001	49	67	Low	Not prognostic	
Stomach cancer	21.29	0.021	38	34	Low/medium	Not prognostic	
Bladder Urothelial Carcinoma	15.46	0.15	5	4	Low/medium	Not prognostic	

## Data Availability

The original contributions presented in this study are included in the article/Supplementary Material. Further inquiries can be directed to the corresponding author.

## Supplementary Materials

The following supporting information can be downloaded at: https://www.mdpi.com/article/10.3390/jcm14145139/s1, Figure S1 mRNA expression of (A) HIF1α, (B) MAPK12 and (C) TGFβ1 in COAD, CESC, LUAD and PAAD compared to normal tissue; Figure S2 Analysis of the risk involved with the expression of CD318 by Hazard Ratio (HR). The data indicate that PAAD and LUAD have a significantly increased risk, while COAD showed a decreased risk with the expression of CD318.
