# Supplementary material for: Differential Role of CD318 in Tumor Immunity Affecting Prognosis in Colorectal Cancer Compared to Other Adenocarcinomas
Source: J Clin Med. 2025 Jul 19;14(14):5139. doi: 10.3390/jcm14145139 (PMC12295231; doi:10.3390/jcm14145139)
Supplement: Supplementary file 1 [file jcm-14-05139-s001.zip › jcm-3729089-supplementary.pdf]

Supplementary Figure1

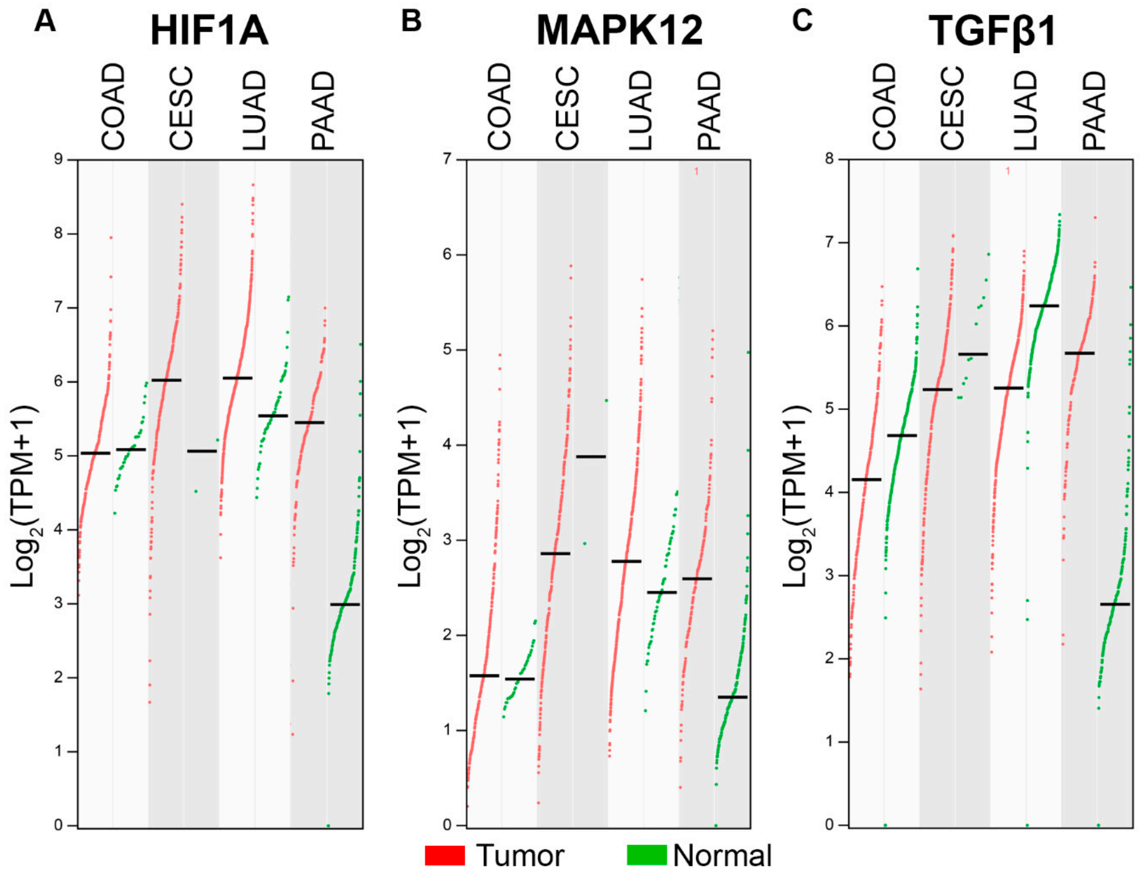

Suppl. Fig. 1 mRNA expression of (A) HIF1α, (B) MAPK12 and (C) TGFβ1 in COAD, CESC, LUAD and PAAD compared to normal tissue.

Note: PAAD data for normal tissue is obtained from both TCGA and GTEx data due to the low sample no. available in TCGA data.

Supplementary Figure2

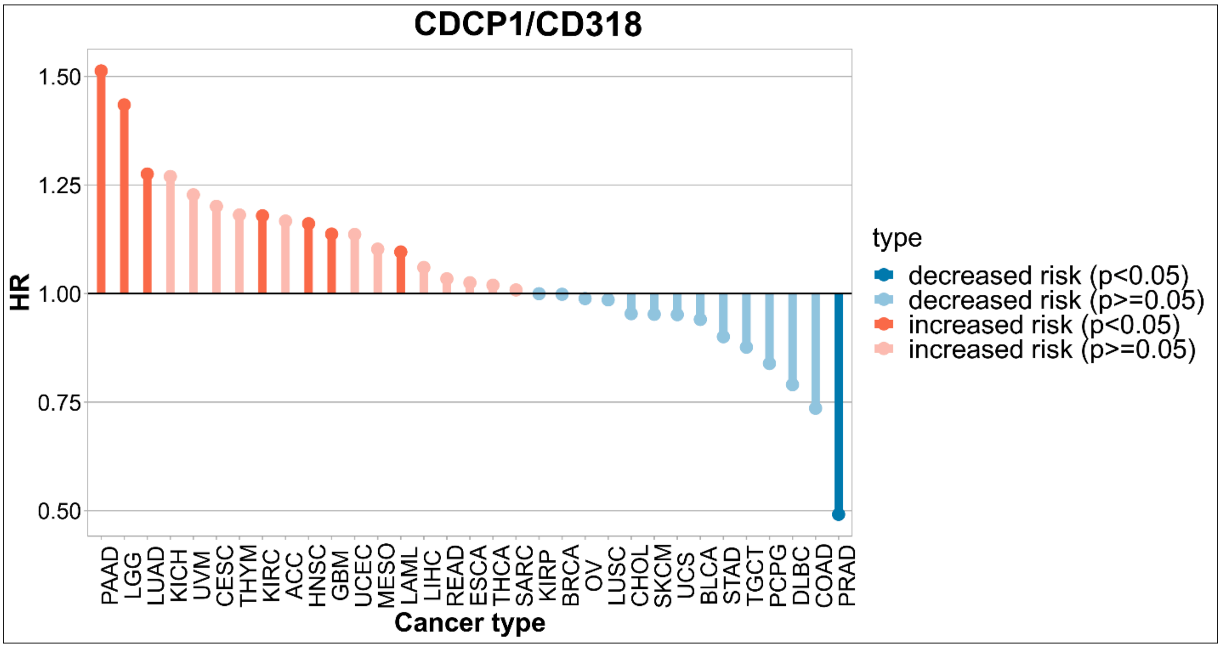

Suppl. Fig. 2 Analysis of the risk involved with the expression of CD318 by Hazard Ratio (HR). The data indicate that PAAD and LUAD have a significantly increased risk, while COAD showed a decreased risk with the expression of CD318.
